# Multifactorial influences on musical creativity: an integrated perspective on cognitive and social capital factors

**DOI:** 10.3389/fpsyg.2025.1585658

**Published:** 2025-08-12

**Authors:** Shaohua Ben, Wenjun Zheng, Xu Li

**Affiliations:** ^1^School of Finance and Economics, Guangxi Science & Technology Normal University, Liuzhou, China; ^2^Faculty of Social Sciences & Liberal Arts, UCSI University, Kuala Lumpur, Malaysia; ^3^School of Physical Education, Chongqing University of Posts and Telecommunications, Chongqing, China

**Keywords:** social capital, creative self-efficacy, musical creativity, musical aesthetic ability, creative thinking

## Abstract

**Introduction:**

Musical creativity is a crucial aspect of music education and innovation. This study has aimed to systematically examine its key determinants, with particular attention to cognitive and social capital mechanisms.

**Methods:**

Grounded in the componential theory of creativity, social capital theory, social cognitive theory, this study has developed a conceptual framework incorporating five explanatory variables. It has employed partial least squares structural equation modeling (PLS-SEM) to analyze data from 962 university students.

**Results:**

The findings have revealed that social capital has exerted both direct effect and moderating effect on creative self-efficacy. While creative thinking has emerged as the strongest antecedent, its influence on musical creativity has diminished under the moderation of social capital. In contrast, musical aesthetic ability, though the weakest antecedent, has demonstrated a significant effect on musical creativity when moderated by social capital. Furthermore, creative self-efficacy has served as a mediating factor in the musical creativity model.

**Conclusion:**

This study has highlighted the complex interplay between cognitive and social capital in shaping musical creativity, thereby enriching its theoretical foundation. The findings have offered novel insights and practical implications for music education by equipping educators with strategies to cultivate students' creative potential and encourage innovation in higher education.

## 1 Introduction

Musical creativity has served as a fundamental catalyst for artistic development, encompassing core activities such as composition, improvisation, performance, listening, and musical analysis (Bashwiner et al., 2020). It has empowered individuals to generate original musical ideas, respond adaptively to dynamic musical contexts, and engage in reflective, personalized music-making (Burnard, 2012). Beyond its artistic value, musical creativity has contributed to broader developmental outcomes, including enhanced cognitive flexibility, emotional expression, and psychological wellbeing (Welch and Ockelford, 2015). Consequently, it has increasingly been recognized as a vital competency for 21st-century learners navigating complex, technology-driven cultural landscapes.

From a psychological perspective, musical creativity is not merely a talent but a skill that can be cultivated through systematic intervention (Craft, 2005). Although society and the educational community have increasingly recognized the importance of creativity in personal development and education, contemporary music education has often failed to effectively stimulate and nurture students' creativity in practice (Lasky and Yoon, 2020; Welch, 2012). Research has suggested that college music courses should serve as ideal environments for fostering students' creativity. However, many traditional music education models have overemphasized technical training while neglecting creative expression, thereby marginalizing creativity within the curriculum (Jackson and Shaw, 2006; Marquis et al., 2017; Piazza and Talbot, 2021).

The componential theory of creativity has been regarded as the most suitable theoretical framework for music research (Huovinen, 2021). It has posited that creativity is influenced by social capital, creative thinking, and domain-specific expertise (Amabile, 1988; Chin et al., 2018; Özgenel and Çetin, 2017). Although cognitive and social capital factors have been considered critical to creative development, few studies have examined their dynamic interplay within the context of musical creation (Schiavio et al., 2022). Empirical findings have shown that creative self-efficacy has exerted a stronger influence on creativity than creative thinking (Huang et al., 2020). Nevertheless, a comprehensive understanding of how these cognitive and social capital mechanisms interact to foster musical creativity has remained insufficient.

Despite the growing interest in musical creativity, research in this area has remained fragmented and has often lacked an integrative perspective (Burnard, 2012; Barrett et al., 2012; Odena and Welch, 2009; Ozenc-Ira, 2023). Many studies have isolated specific elements of creativity (e.g., cognitive processes, emotional engagement, or motivational drivers) without adequately examining how these components interact within complex, interdisciplinary frameworks (Hargreaves et al., 2012). This lack of integration has created a significant gap in the literature, particularly concerning how multi-level interactions among cognitive, emotional, and social capital factors have contributed to creative outcomes in music (Brattico et al., 2013; Brattico and Varankaite, 2019; Schindler et al., 2017; Zentner et al., 2008). This study has aimed to address this gap by synthesizing existing theory and empirical research to develop an interdisciplinary framework that integrates psychology, music education, and cognitive science. In doing so, it has sought to advance psychological theories of musical creativity while offering practical insights for music educators. The findings are expected not only to enhance music education practices but also to contribute to a more nuanced understanding of the psychological mechanisms underpinning musical creativity, ultimately supporting the cultivation of students' creative potential in a more holistic manner.

## 2 Theoretical background

(Bandura 1986) has emphasized that self-efficacy plays a central role in creative behavior. Applied to creativity, creative self-efficacy has represented individuals' confidence in their ability to generate creative outcomes, serving as a key psychological mechanism that links social influences to performance (Bandura, 1997; Tierney and Farmer, 2002). From the perspective of Social Capital Theory, trust-based relationships and shared norms have enhanced individuals' belief in their creative capacity, thereby enabling more effective engagement in creative tasks (Coleman, 1988; Woolcock and Narayan, 2000; Woolcock, 2002). Meanwhile, the Componential Theory of Creativity has proposed that both social capital and creative self-efficacy are essential for musical creative performance, particularly when supported by domain-relevant skills and a conducive social environment (Amabile, 1983, 2013; Torrance, 1988). Accordingly, creative self-efficacy has emerged as a point of theoretical convergence, mediating or moderating the influence of social capital (external condition) and creative thinking or musical aesthetic ability (internal processes) on musical creativity. As depicted in the conceptual model, creative self-efficacy has functioned as the sole mediating construct connecting social capital, creative thinking, and musical aesthetic ability with musical creativity. This reflects a social-cognitive pathway that has integrated external resources and internal processes.

## 3 Literature review and research hypotheses

Social capital has highlighted how relationships grounded in trust and shared norms have contributed to personal growth and capability development (Coleman, 1988; Nahapiet and Ghoshal, 1998; Woolcock and Narayan, 2000). Furthermore, it has provided emotional support and instrumental resources that have strengthened creative self-efficacy and engagement (Woolcock, 2002; Williams, 2006). In educational contexts, strong peer relationships and collaborative networks have been shown to enhance creative learning and foster students' sense of efficacy (Chiu et al., 2006; Chow and Chan, 2008).

Musical creativity has been understood as a process influenced by expertise (i.e., deep and rich knowledge), creative thinking (i.e., problem-solving methods), and social capital (i.e., passion for the task), all of which have directly shaped creative musical outcomes (Amabile, 1988; Webster, 1990; Sternberg, 2003; Dilekçi and Karatay, 2023; Durnali et al., 2023). (Hickey and Webster 2001, p. 11) has defined musical creativity as “the engagement of the mind in the active, structured process of thinking in sound to produce some product that is new for the creator.” However, little empirical research has been conducted on the application of the Componential Theory of Creativity within the field of music. Musical creativity has remained a complex and multidimensional phenomenon, involving the synergy of various cognitive and affective factors (Palhares et al., 2022).

**Figure 1:**
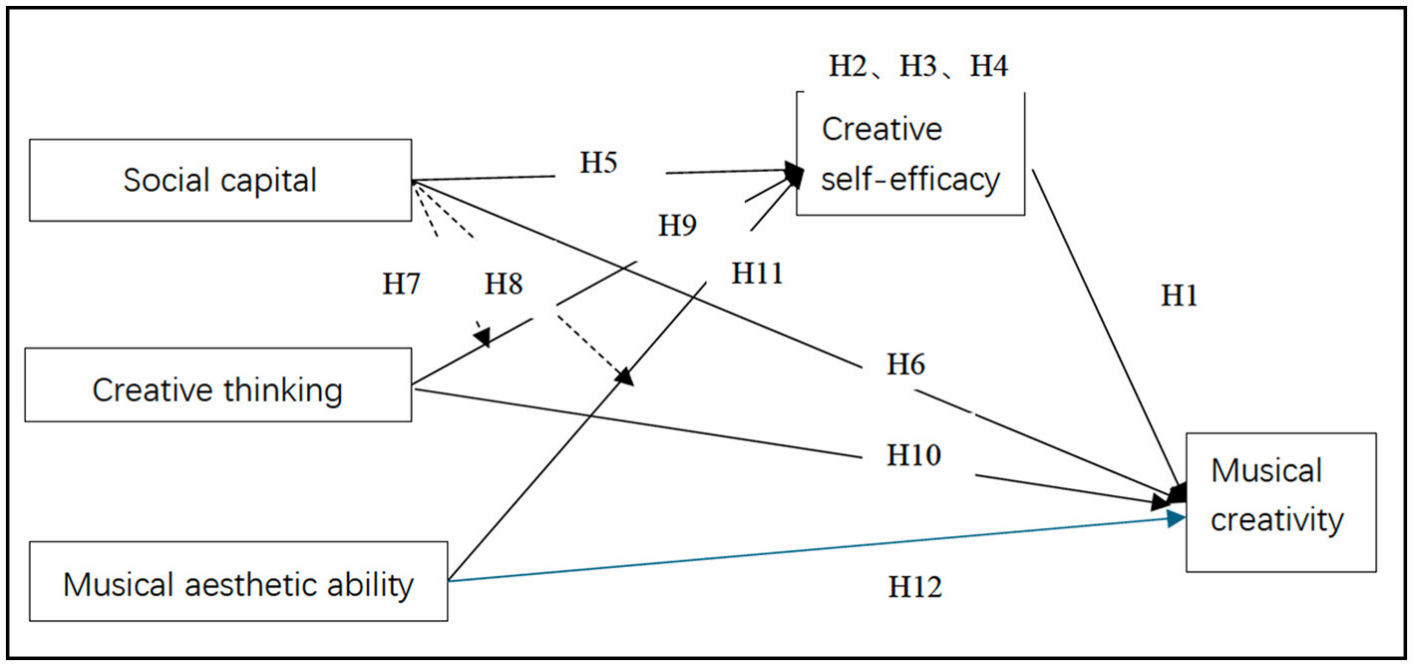
Research model and hypotheses. MC, Musical creativity; CT, Creative thinking; MAA, Musical aesthetic ability; SC, Social capital; CSE, Creative self-efficacy.

The present study has constructed a model that has integrated social capital, creative thinking, musical aesthetic ability, and creative self-efficacy to predict musical creativity. As illustrated in Figure 1, creative self-efficacy has been positioned as the sole mediating variable, reflecting the explanatory power of Social Cognitive Theory in bridging the Componential Theory of Creativity and Social Capital Theory. Additionally, the model has examined the moderating effects of social capital on the relationships between cognitive components—namely, creative thinking and musical aesthetic ability—and creative self-efficacy (Kritsotakis et al., 2008; Perez Fernandez et al., 2024).

### 3.1 The internal process

#### 3.1.1 Creativity self-efficacy

Creative self-efficacy has referred to an individual's belief in their capacity to produce creative outcomes, drawing from Bandura's (1997) general theory of self-efficacy and subsequently adapted to creativity by Tierney and Farmer (2002). It has influenced individuals' motivation, task engagement, and persistence in creative endeavors. Empirical studies have consistently demonstrated that creative self-efficacy is positively associated with creativity-related outcomes. For instance, Bicer et al. (2020) have emphasized the significance of a strong sense of creative self-belief, while Qiang et al. (2020) have identified it as a predictor of scientific and musical creativity among high school students. Individuals with higher creative self-efficacy have typically exhibited greater resilience, a lower adherence to conventional norms, and a stronger inclination toward innovative thinking and problem-solving (Huang et al., 2020). Based on the preceding literature and theoretical discussion, this study has proposed the following hypotheses (see Figure 1).

**H1:** Creativity self-efficacy has a significant effect on musical creativity.

Social capital has enhanced individual agency and psychological development by facilitating access to supportive social environments (Nahapiet and Ghoshal, 1998; Woolcock and Narayan, 2000; Chetty et al., 2022). Within this framework, creative self-efficacy has emerged as a central psychological mechanism through which social structures have exerted their influence. Self-efficacy has consistently been identified as a mediator in the link between social factors and personal outcomes, suggesting that confidence in one's abilities has bridged the gap between social support and individual performance (Kritsotakis et al., 2008). Specifically, creative self-efficacy has been empirically demonstrated to mediate the relationship between social capital and musical creativity, affirming its role as a transformative conduit between social environments and creative expression (Perez Fernandez et al., 2024). Based on the preceding literature and theoretical rationale, this study has proposed the following hypotheses (see Figure 1).

**H2:** Creative self-efficacy has a significant mediating effect on social capital and musical creativity.

Furthermore, findings regarding the relationship between creative self-efficacy and creative thinking have remained mixed. While some studies have reported positive effects on ideation and fluency (Qiang et al., 2020; Farmer and Tierney, 2017), others have found no significant correlation or have even yielded contradictory results (Jaussi et al., 2007). Haase et al. (2018) have suggested that these inconsistencies may have stemmed from variations in the design of creativity tasks. Based on the preceding literature and theoretical discussion, this study has proposed the following hypotheses (see Figure 1).

**H3:** Creative self-efficacy has a significant mediating effect on creative thinking and musical creativity.

Recent work by Chen et al. (2024) has highlighted a bidirectional relationship between creative self-efficacy and musical aesthetic ability, consistent with Csikszentmihalyi's (1990) theory of flow, which has asserted that musical aesthetic ability strengthens when individuals believe in their creative capabilities. This perspective has also aligned with Van den Broeck et al. (2016), who have argued that musical aesthetic ability fosters intrinsic enjoyment and sustained engagement, even when individuals encounter creative challenges. Based on the preceding literature and conceptual rationale, this study has proposed the following hypotheses (see Figure 1).

**H4:** Creative self-efficacy has a significant mediating effect on musical aesthetic ability and musical creativity.

### 3.2 The external constructs

#### 3.2.1 Social capital

Social capital, defined as the resources embedded within social networks, has played a central role in the development of individual cognitive and psychological capacities (Coleman, 1988). It has enabled individuals to access constructive feedback, emotional encouragement, and shared norms that have collectively strengthened their self-belief. Empirical studies have consistently demonstrated that robust social networks have enhanced individuals' perceived competence and confidence (Woolcock, 2002; Williams, 2006; Portes, 2024). Within educational contexts, social trust and peer interaction have fostered students' creative self-efficacy by shaping collaborative and supportive learning environments (Chiu et al., 2006; Chow and Chan, 2008). Based on this body of evidence, the following hypothesis has been proposed (see Figure 1).

**H5:** Social capital has a significant effect on creative self-efficacy.

Social capital has also directly facilitated creative expression. According to the relational view of social capital, it has served as a foundation for trust-based knowledge exchange, thereby stimulating innovative thinking (Nahapiet and Ghoshal, 1998). In the context of music education, access to diverse social interactions has provided not only emotional support but also creative inspiration and opportunities for collaboration (Woolcock and Narayan, 2000). This connection has proven particularly significant in artistic domains, where co-creation and mutual appreciation have enhanced originality. Accordingly, the following hypothesis has been proposed (see Figure 1).

**H6:** Social capital has a significant effect on musical creativity.

Although creative thinking has served as a primary driver of creativity, its impact has often been shaped by the surrounding social environment (Hickey and Webster, 2001). Social capital has been shown to either facilitate or constrain the realization of creative potential, depending on the levels of trust, shared norms, and access to supportive structures embedded in a given context (Woolcock, 2002). In socially enriched environments, individuals have been more likely to receive constructive feedback, recognition, and encouragement, all of which have enhanced the expression of their creative thinking. Moreover, social-contextual conditions have significantly influenced the effectiveness of psychological traits such as creativity, underscoring the importance of simultaneously considering both personal dispositions and environmental influences in creativity research (Kritsotakis et al., 2008; Durnali et al., 2023). Accordingly, the following hypothesis has been proposed (see Figure 1).

**H7:** Social capital has a significant moderating effect on creative thinking and musical creativity.

Musical aesthetic ability has been widely recognized as a foundational dimension of musical creativity (Amabile, 1988; Fingerhut et al., 2021; Clemente et al., 2022). However, its influence on creative outcomes has likely been shaped by social capital factors (Wahed et al., 2021). As Woolcock (2002) has suggested, individuals have been more capable of transforming aesthetic perception into creative performance when embedded in supportive social networks. Trust, shared values, and peer appreciation for artistic expression have strengthened the link between aesthetic sensitivity and creative output (Williams, 2006; Chow and Chan, 2008). Accordingly, the following hypothesis has been proposed (see Figure 1).

**H8:** Social capital has a significant moderating effect on musical aesthetic ability and musical creativity.

#### 3.2.2 Creative thinking

Creative thinking has been widely recognized as a foundational skill in the creative process. According to Torrance (1988), it has involved problem-solving, hypothesizing, generating novel ideas, and presenting innovative solutions. Prior empirical studies have demonstrated a positive association between creative self-efficacy and the development of creative thinking (Huang et al., 2020; Dilekçi and Karatay, 2023). Webster (1990) has defined creative thinking in music as a dynamic psychological process driven by internal musical abilities and external stimuli, culminating in the creation of a new musical product. Creativity has been conceptualized as a latent personal trait that can be observed through performance in structured creative thinking tasks (Kupers et al., 2019; Kupers and van Dijk, 2020; Dilekçi and Karatay, 2023; Durnali et al., 2023). Accordingly, the following hypothesis has been proposed (Figure 1).

**H9:** Creative thinking has a significant effect on creativity self-efficacy.
**H10:** Creative thinking has a significant effect on musical creativity.

#### 3.2.3 Musical aesthetic ability

Musical aesthetic ability has been defined as the capacity to perceive, understand, evaluate, appreciate, and create beauty in music (Dan et al., 2021). Prior studies have highlighted its significance in fostering creative development. For instance, Raikou (2016) has demonstrated that aesthetic sensitivity contributes meaningfully to the enhancement of creativity. Additionally, higher levels of aesthetic appreciation have been associated with positive emotional experiences and increased creative output. Specifically, positive emotions have shown strong correlations with creativity, whereas negative emotions have not exhibited such associations (Świa̧tek et al., 2023a,b). One widely used method to assess creative self-efficacy has involved asking individuals to reflect on their experiences and emotional states during creative engagement. Reports of curiosity, interest, enjoyment, and similar positive feelings have been interpreted as indicators influenced by creative self-efficacy (Sarasso et al., 2021; Fishbach and Woolley, 2022; Clemente et al., 2022). These findings have indirectly confirmed the linkage between musical aesthetic ability and creative self-efficacy. Accordingly, the following hypothesis has been proposed (Figure 1).

**H11:** Musical aesthetic ability has a significant effect on creativity self-efficacy.
**H12:** Musical aesthetic ability has a significant effect on musical creativity.

## 4 Methods

### 4.1 Participants and data collection

The participants in this study were university students from China. Data were collected through an online survey distributed via the WeChat platform. Respondents provided demographic information and completed a series of creativity-related scales and assessments. A total of 962 valid questionnaires were collected, all of which contained complete responses. Among the participants, 525 were female (54.6%) and 437 were male (45.4%). The majority of respondents (*n* = 627, 65.2%) were between 18 and 25 years old.

### 4.2 Measures

This study employed previously validated measurement scales, with item wording adapted to suit the specific research context. A 10-point semantic differential scale was used to assess participants' level of agreement with each item, ranging from 1 (strongly disagree) to 10 (strongly agree). The hypothesized model included three exogenous variables (social capital, creative thinking, and musical aesthetic ability), one mediating variable (creative self-efficacy), and one endogenous variable (musical creativity).

#### 4.2.1 Social capital

This study used 30 items from social capital scale (Onyx and Bullen, 2000). With acceptable internal consistency (α = 0.76). Social capital scale to measure university students' perspective of music. Sample items included “I like the intensity of the experience that music gives me” and “Being similar to my peers in terms of musical tastes helps us connect better.”

#### 4.2.2 Creative thinking

The structure was assessed using the Marmara Creative Thinking Dispositions Scale (Özgenel and Çetin, 2017), an instrument containing 24 items (α = 0.91). The instrument has been extensively validated. It consists of six dimensions: Innovation search (α = 0.84), courage (α = 0.67), self-discipline (α = 0.71), inquisitive (α = 0.67), doubt (α = 0.71), and flexibility (α = 0.61). The scores for each dimension were calculated to indicate the students' creative thinking.

#### 4.2.3 Musical aesthetic ability

This study assessed university students' musical aesthetic ability through two dimensions, perception, and appreciation, based on theories of art education (Dan et al., 2021). Students were asked to complete a short-written test, and the final score was used to measure the construct.

#### 4.2.4 Creative self-efficacy

To measure teachers' creative self-efficacy, we used a three-item scale developed by Tierney and Farmer (2002) (α = 0.86). The items were: (1) “I am good at coming up with new ideas,” (2) “I have a knack for solving problems creatively,” and (3) “I have confidence in my ability to be creative.” Respondents rated each item on a 5-point Likert scale ranging from 1 (strongly disagree) to 5 (strongly agree). The final CSE score was computed by averaging the three items, with higher scores indicating greater creative self-efficacy.

#### 4.2.5 Musical creativity

Five items were adapted from Osmani et al. (2022), which described individuals' subjective ratings of their creativity abilities (CR = 0.756). Example item: I look for new solutions even if there is no clear need.

### 4.3 Data analysis

This study employed Structural Equation Modeling (SEM) as the primary analytical methodology. Given the study's focus on assessing the relationships among multiple constructs, Partial Least Squares Structural Equation Modeling (PLS-SEM) was deemed the most appropriate analytical approach (Hair et al., 2017). As a second-generation regression technique, PLS-SEM enables the simultaneous estimation of both measurement and structural models, providing a robust means of examining complex variable relationships (Hair et al., 2017). Following Hair et al.'s (2017) guidelines, the analysis proceeded in two stages. First, the measurement model was assessed to establish construct reliability and validity. Then, the structural model was evaluated to test the hypothesized relationships among variables. All statistical analyses were conducted using SmartPLS version 4.1.

## 5 Results

### 5.1 Measurement model

Assessment of the measurement model was based on four key criteria: indicator reliability, internal consistency reliability, convergent validity, and discriminant validity (Hair et al., 2017).

To evaluate indicator reliability, standardized factor loadings of all items were examined. Loadings above 0.40 were considered acceptable, following Hair et al. (2017). Four items with low loadings were removed, and the remaining indicators demonstrated satisfactory reliability.

Internal consistency was assessed using Cronbach's alpha and composite reliability (CR). As shown in Table 1, all constructs exceeded the recommended threshold of 0.70, indicating acceptable structural reliability.

**Table 1:** Reliability and validity results.

**Constructs**	**Cronbach's alpha**	**Composite reliability**	**AVE**
Creative self-efficacy (CSE)	0.818	0.892	0.734
Musical aesthetic ability (MAA)	0.942	0.951	0.659
Social capital (SC)	0.966	0.968	0.504
Creative thinking (CT)	0.961	0.964	0.521
Musical creativity (MC)	0.933	0.934	0.788

AVE, Average Variance Extracted.

Convergent validity was confirmed by calculating the Average Variance Extracted (AVE), which should exceed 0.50. Table 1 indicates that all AVE values met this threshold, with the minimum AVE being 0.504, suggesting that each construct accounted for at least 50% of the variance in its indicators.

Discriminant validity was assessed using both the Fornell–Larcker criterion and the Heterotrait–Monotrait ratio (HTMT). According to Fornell and Larcker (1981), the square root of the AVE for each construct should be greater than its correlations with other constructs. After evaluating indicator performance, 22 items were removed. As shown in Table 2, the remaining constructs met the Fornell–Larcker criterion. HTMT values were also computed to assess discriminant validity. As recommended by Henseler et al. (2015), all HTMT values were below the conservative threshold of 0.90 (Table 3), thereby confirming satisfactory discriminant validity.

**Table 2:** Fornell-Larcker criterion.

	**MC**	**CSE**	**CT**	**MAA**	**SC**
1. MC	0.917				
2. CSE	0.467	0.857			
3. CT	0.596	0.541	0.722		
4. MAA	0.449	0.449	0.554	0.812	
5. SC	0.584	0.572	0.837	0.623	0.710

MC, Musical creativity; CT, Creative thinking; MAA, Musical aesthetic ability; SC, Social capital; CSE, Creative self-efficacy.

**Table 3:** Heterotrait-monotrait ratio (HTMT).

	**MC**	**CSE**	**CT**	**MAA**	**SC**
1. MC					
2. CSE	0.529				
3. CT	0.622	0.609			
4. MAA	0.473	0.568	0.580		
5. SC	0.609	0.643	0.869	0.653	

MC, Musical creativity; CT, Creative thinking; MAA, Musical aesthetic ability; SC, Social capital; CSE, Creative self-efficacy.

### 5.2 Structural model

The structural model (i.e., path model) was evaluated following the criteria proposed by Hair et al. (2017), including: (1) collinearity assessment, (2) the significance and relevance of structural relationships, (3) the model's explanatory power (R^2^), and (4) predictive relevance (Q^2^). To assess the robustness of the model, a bootstrapping procedure was conducted, and both direct and indirect effects were examined.

First, collinearity was evaluated using the Variance Inflation Factor (VIF), a standard metric for detecting multicollinearity. All constructs exhibited VIF values below the threshold of 5, indicating no significant multicollinearity issues (Hair et al., 2017).

Second, the R^2^ values for the endogenous variables are reported in Table 4. These values reflect the proportion of variance explained by the exogenous variables. According to Hair et al. (2017), R^2^ values of 0.26 to 0.50 indicate moderate explanatory power. In this study, musical creativity (R^2^ = 0.402) and creative self-efficacy (R^2^ = 0.378) fall within this range, suggesting a moderate level of model fit.

**Table 4:** R^2^ and Q^2^ of endogenous variables.

**Endogenous variable**	**Explained variance (R2)**	**Prediction relevance (Q2)**
Musical creativity (MC)	0.402	0.381
Creative self-efficacy (CSE)	0.378	0.367

Third, predictive relevance was assessed using the Q^2^ value, obtained via blindfolding procedures. Based on Hair et al.'s (2017) guidelines, Q^2^ values above 0.25 indicate moderate predictive relevance, while values above 0.50 suggest high predictive relevance. Both creative self-efficacy and musical creativity yielded Q^2^ values above 0.25, confirming moderate predictive relevance (see Table 4).

To test the proposed hypotheses and structural relationships, standardized path coefficient analyses were conducted (Table 5). The results revealed that creative thinking exerted the strongest positive influence on musical creativity (β = 0.315, *p* < 0.05), followed by social capital (β = 0.180, *p* < 0.05), creative self-efficacy (β = 0.150, *p* < 0.05), and musical aesthetic ability (β = 0.088, *p* < 0.05). These findings suggest that all four variables significantly contribute to musical creativity. Accordingly, hypotheses H1, H6, H10, and H12 were supported.

**Table 5:** Hypotheses testing.

**Ha**	**Path**	**β**	**(95%)CI**	***p***	**Result**
H1	CSE → MC	0.150	(0.075, 0.231)	0.000	Supported
H2	SC → CSE → MC	0.043	(0.018, 0.080)	0.006	Supported
H3	CT → CSE → MC	0.029	(0.008, 0.060)	0.030	Supported
H4	MAA → CSE → MC	0.030	(0.025, 0.119)	0.005	Supported
H5	SC → CSE	0.286	(0.156, 0.417)	0.000	Supported
H6	SC → MC	0.180	(0.109, 0.348)	0.002	Supported
H7	SC ^*^ CT → CSE	−0.062	(−0.151, 0.035)	0.190	Not supported
H8	SC ^*^ MAA → CSE	0.105	(0.016, 0.191)	0.018	Supported
H9	CT → CSE	0.193	(0.049, 0.323)	0.006	Supported
H10	CT → MC	0.315	(0.211, 0.460)	0.000	Supported
H11	MAA → CSE	0.198	(0.111, 0.288)	0.000	Supported
H12	MAA → MC	0.088	(0.013, 0.056)	0.024	Supported

MC, Musical creativity; CT, Creative thinking; MAA, Musical aesthetic ability; SC, Social capital; CSE, Creative self-efficacy.

Regarding the antecedents of creative self-efficacy—the study's main mediating construct—social capital emerged as the strongest predictor (β = 0.286, *p* < 0.05), followed by musical aesthetic ability (β = 0.198, *p* < 0.05) and creative thinking (β = 0.193, *p* < 0.05). These results indicate that these three variables play significant roles in enhancing students' belief in their creative capacities. Creative self-efficacy was also found to mediate the effects of social capital, creative thinking, and musical aesthetic ability on musical creativity. Therefore, hypotheses H2, H3, H4, H5, H9, and H11 were supported (Table 5).

**Figure 2:**
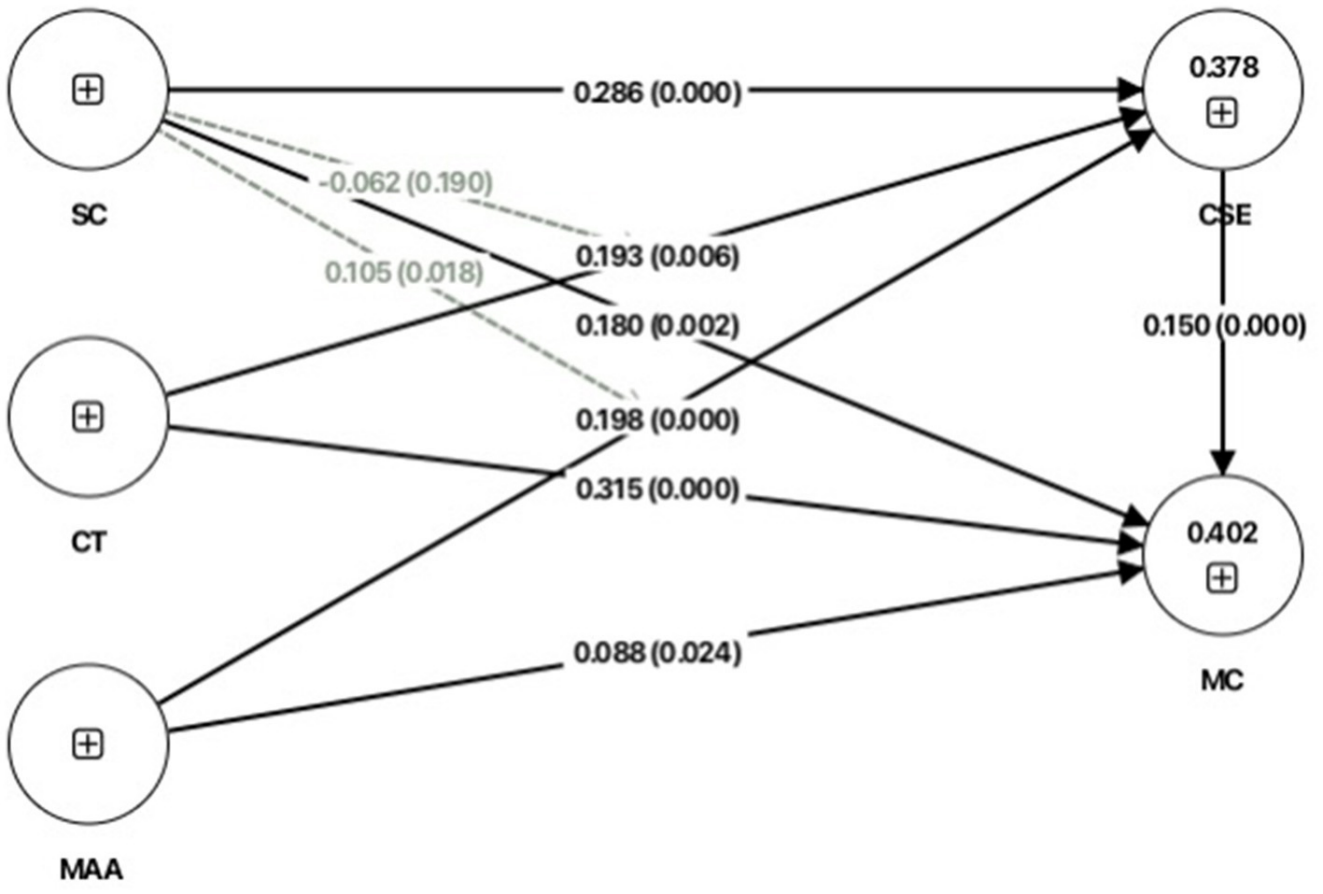
PLS-SEM path coefficients and significance testing results of the structural model. MC, Musical creativity; CT, Creative thinking; MAA, Musical aesthetic ability; SC, Social capital; CSE, Creative self-efficacy.

Additionally, the study examined the moderating role of social capital. Results showed that social capital significantly moderated the relationship between musical aesthetic ability and musical creativity (β = 0.105, *p* < 0.05), but did not significantly moderate the relationship between creative thinking and musical creativity (β = −0.062, *p* > 0.05). Thus, hypothesis H8 was supported, whereas H7 was not (Table 5). Figure 2 presents the structural model of the study, illustrating all validated paths and variable relationships.

## 6 Discussion

This study investigated the underlying mechanisms of musical creativity by extending the componential model of creativity to incorporate both cognitive and social capital factors. The findings offer meaningful contributions to the literature on music education and creativity by revealing how internal traits and external conditions interact to shape creative outcomes. Four key discussions emerged: (1) Discussion 1 (H1, H2, H3, H4), Creative self-efficacy functioned as a mediator between the three antecedents—social capital, creative thinking, and musical aesthetic ability—and musical creativity, confirming its central role in the creative process. (2) Discussion 2 (H5, H6), social capital not only exerts a direct influence on creative self-efficacy but also plays a moderating role, enhancing the impact of other cognitive factors on creative outcomes. (3) Discussion 3 (H7, H9, H10), creative thinking emerged as the strongest predictor of musical creativity. However, its positive effect was not significant when social capital was introduced as a moderator, suggesting no significant interaction between creative thinking and social capital among university students. (4) Discussion 4 (H8, H11, H12), Musical aesthetic ability showed the weakest direct effect on musical creativity. Nevertheless, its relationship with musical creativity became significant under the moderating effect of social capital, highlighting the role of social context in amplifying aesthetic perception.

Regarding Discussion 1 (H1, H2, H3, H4), the results demonstrate that creative self-efficacy functions as a key mediating variable, underscoring both its theoretical relevance in shaping indirect pathways and its practical significance as a direct driver of musical creativity. First, the mediating effect of creative self-efficacy aligns with Bandura's (1997) self-efficacy theory, which posits that self-belief is a central mechanism through which external factors influence performance. In line with this, social capital enhances creative performance by strengthening individuals' creative self-efficacy (Chiu et al., 2006). Second, the influence of creative thinking on musical creativity is also mediated by creative self-efficacy, as it boosts individuals' confidence in successfully engaging in creative tasks, thereby motivating greater innovation (Zeng et al., 2022). Third, musical aesthetic ability fosters musical creativity indirectly by enhancing creative self-efficacy, affirming that aesthetic experience and sensitivity act as psychological resources that promote creative output through strengthened efficacy beliefs (Chen et al., 2001; Smith et al., 2020). Collectively, these findings reinforce the central role of creative self-efficacy in the psychological mechanisms underlying musical creativity (Amabile and Pratt, 2016). Result 4 reveals that creative self-efficacy serves as a pure mediator, playing a dual role by linking key antecedents to musical creativity. Theoretically, it captures both internal psychological processes and a core cognitive resource for creativity. Statistically, it enhances the explanatory power of the model, and practically, it represents a strategic focal point for creativity-oriented educational interventions.

Regarding discussion 2 (H5, H6), This study confirms that social capital plays a multifaceted and influential role in music education by both directly and indirectly shaping students' creative outcomes. Specifically, social capital was found to have a significant direct effect on creative self-efficacy (H5 supported) and musical creativity (H6 supported). According to social capital theory (Coleman, 1988), access to trust-based relationships and supportive networks not only fosters engagement but also enhances individuals' belief in their creative capabilities (Woolcock and Narayan, 2000). This finding aligns with prior research suggesting that students with higher levels of social capital tend to exhibit stronger creative self-efficacy (Beghetto and Kaufman, 2014). Moreover, social capital acts as a moderator, strengthening the effect of musical aesthetic ability on creative self-efficacy. In line with earlier studies (Kritsotakis et al., 2008; Chow and Chan, 2008; Perez Fernandez et al., 2024), this study further demonstrates that individuals embedded in rich social networks are more likely to engage deeply in musical creation, invest greater cognitive resources, and produce more original and high-quality outputs. Thus, social capital serves a dual function, directly promoting musical creativity and amplifying the impact of other antecedents, making it a critical enabler in the development of creative potential.

Regarding Discussion 3 (H7, H9, H10), the findings reveal that creative thinking exerts a significant positive effect on creative self-efficacy (H9 supported), consistent with Bandura's (1997) self-efficacy theory, which posits that individuals strengthen their belief in their capabilities through evaluative and reflective cognitive activities. Creative thinking also significantly enhances musical creativity (H10 supported), corroborating prior research that highlights its central role in driving creative output in music (Sternberg, 2006; Amabile and Pratt, 2016). However, social capital was not found to significantly moderate the relationship between creative thinking and creative self-efficacy (H7 not supported), suggesting that the influence of creative thinking on self-efficacy remains stable across varying levels of social capital. This may be attributed to the inherent nature of creative thinking as a stable cognitive process, relatively unaffected by fluctuations in external contextual factors (Hennessey and Amabile, 2010; Beghetto and Kaufman, 2014). Often operationalized as divergent thinking, creative thinking emphasizes the capacity to generate multiple, novel, and flexible ideas (Guilford, 1967; Sternberg and Lubart, 1999; Hickey and Webster, 2001), a process less reliant on social feedback or group-based support. Consequently, social capital does not significantly moderate the relationship between creative thinking and musical creativity. These findings suggest that the development of creative thinking is largely independent of social capital, implying that even substantial investment in social or educational infrastructure may not directly enhance students' creative thinking unless cognitive mechanisms are specifically targeted through instructional design and pedagogical intervention.

Regarding Discussion 4 (H8, H11, H12), the results indicate that musical aesthetic ability has a significant positive effect on creative self-efficacy (H11 supported). This aligns with Csikszentmihalyi and Robinson's (1990) findings, which suggest that the positive emotions and perceptual experiences evoked during aesthetic engagement enhance individuals' belief in their creative capacities, thereby boosting creative self-efficacy. Musical aesthetic ability also significantly predicts musical creativity (H12 supported), reinforcing prior research that identifies aesthetic sensitivity as a foundational precursor to creative output (Zentner et al., 2008; Brattico et al., 2013; Świa̧tek et al., 2023b). Moreover, this study confirms the moderating role of social capital in the relationship between musical aesthetic ability and creative self-efficacy (H8 supported), indicating that individuals with higher levels of social capital benefit more strongly from the influence of aesthetic experience on self-belief. This finding supports Amabile and Pratt's (2016) theoretical proposition that social capital can amplify the translation of individual abilities and experiences into enhanced self-efficacy, which in turn fosters creative performance. Taken together, Result 3 provides compelling support for positioning musical aesthetic ability as an essential curricular objective within music education syllabi.

## 7 Limitations and future research

This study recognizes the limitations of its scope, primarily due to restricted sample size and geographic representation. Future research needs to be conducted with a broader sample, including individuals from different ages, socioeconomic backgrounds, and ethnicities, to obtain more generalizable results. Additionally, consideration should be given to the use of multiple data collection methods, such as behavioral observations, experimental designs, and physiological measurements, to increase the data's objectivity and comprehensiveness. Finally, the inherent limitations of self-report assessments should be considered as this may be subject to memory errors or the way they perceive social expectations to answer. Future research could combine self-report questionnaires with actual performance tests of musical creativity to confirm the findings and provide greater insight into the gap between subjective assessments and objective measures, increasing the reliability and validity of the data.

## 8 Conclusion

This study reveals the main determinants of musical creativity and the complex mechanisms that influence it through theorizing and empirical analysis. This study proposes and validates a triangular antecedent model centered on social capital, creative thinking, and musical aesthetic ability, systematically revealing the key influencing factors and mechanisms underlying musical creativity. First, the results suggest that social capital not only has positive effect on creative self-efficacy, but also exhibits a moderated effect on creative self-efficacy. Second, creative thinking is identified as the most prominent predictor of musical creativity, However, this moderated effect is not significantly by social capital, suggesting that the impact of creative thinking on musical creativity remains relatively stable across varying levels of social capital. Third, musical aesthetic ability is the lowest factor to affect musical creativity, but the relationship between musical aesthetic ability and musical creativity is significantly moderated by social capital, implying that the contribution of aesthetic perception to creativity may vary depending on contextual social capital. Fourth, creative self-efficacy plays a crucial mediating role in the model, confirming that belief in one's own abilities is a key psychological mechanism that transforms social capital, creative thinking and musical aesthetic ability into musical creative.

Overall, the findings of this study deepen the theoretical understanding of musical creativity by emphasizing the central role of creative self-efficacy and uncovering the differential contextual impact of social capital, creativity thinking, musical aesthetic ability. By examining student social capital from a multidimensional perspective and distinguishing between divergent thinking of creativity thinking and convergent judgment of musical aesthetic ability. These insights can provide a reference for developing targeted support programs aimed at creating a supportive musical environment for university students. These results not only provide important theoretical contributions to the field of musical education and creativity research but also offer valuable insights and implications for other domains of creativity-related inquiry and practice.

## Data Availability

The original contributions presented in the study are included in the article/Supplementary material, further inquiries can be directed to the corresponding authors.
